# Terminal Residue and Dietary Risk Assessment of Atrazine and Isoxaflutole in Corn Using High-Performance Liquid Chromatography–Tandem Mass Spectrometry

**DOI:** 10.3390/molecules28207225

**Published:** 2023-10-23

**Authors:** Junli Cao, Tao Pei, Yonghui Wang, Shu Qin, Yanli Qi, Pengcheng Ren, Jindong Li

**Affiliations:** 1 Shanxi Center for Testing of Functional Agro-Products, Shanxi Agricultural University, No. 79, Longcheng Street, Taiyuan 030031, China; caojunli@sxau.edu.cn (J.C.); w1292692554@163.com (T.P.); 15212426571@163.com (Y.W.); qinshu@sxau.edu.cn (S.Q.); qiyanli0412@126.com (Y.Q.); zoe1946@126.com (P.R.); 2 College of Plant Protection, Shanxi Agricultural University, No. 81, Longcheng Street, Taiyuan 030031, China

**Keywords:** isoxaflutole, atrazine, terminal residues, dietary risk assessment

## Abstract

Isoxaflutole and atrazine are representative pesticides for weed control in corn fields. Formulations containing these two pesticides have been registered in China, and their residues may threaten food safety and human health. In this study, a method for simultaneous determination of isoxaflutole, atrazine, and their metabolites in fresh corn, corn kernels, and corn straw was established based on modified QuEChERS pre-treatment and high-performance liquid chromatography–tandem mass spectrometry (HPLC–MS/MS). The linearity of seven compounds was good (R^2^ ≥ 0.9912), and the matrix effect was 48.5–77.1%. At four spiked levels of 0.01, 0.02, 0.05, and 0.5 mg kg^−1^, all compounds’ average recovery was 76% to 116%, with relative standard deviation (RSD) less than 18.9%. Field experiments were conducted in Liaoning, Heilongjiang, Inner Mongolia, Shanxi, Beijing, and Yunnan provinces to study the terminal residues. The terminal residues of all compounds were below the LOQ (0.01 mg kg^−1^) in fresh corn and corn kernels, and atrazine residues in corn straw ranged from <0.05 mg kg^−1^ to 0.17 mg kg^−1^. Finally, a dietary risk assessment was conducted based on residues from field trials, food consumption, and acceptable daily intake (ADI). For all populations, the chronic dietary risk probability (RQ_c_) of atrazine was between 0.0185% and 0.0739%, while that of isoxaflutole was 0.0074–0.0296%, much lower than 100%. The results may provide scientific guidance for using isoxaflutole and atrazine in corn field ecosystems.

## 1. Introduction

Corn (*Zea mays* L.), also known as maize, is one of the world’s major grains along with rice and wheat, rich in dietary fiber, vitamins, minerals, and other nutrients [1]. Fresh corn and corn kernels are essential to the human diet, and straw can feed livestock and poultry. China is a major corn production and consumption country, with a planting area of 43,355,859 hectares in 2021 (https://www.fao.org/faostat/en/#compare, accessed on 5 May 2023). However, weeds are seriously harmful, resulting in a 30% to 40% reduction in corn yield [2]. For weed control, pesticides are currently the most effective means to reduce crop yield losses and are indispensable for agricultural production [3,4]. However, the widespread and repeated application of pesticides around the world has resulted in pesticide residues and potential risks to humans through the food chain, which has become an issue of general concern in food and the environment [5,6]. More seriously, recent studies have reported that pesticide metabolites are frequently detected and may have higher adverse effects on non-target organisms than parent compounds [7,8]. Therefore, more attention should be paid to residue analysis and dietary intake risk of pesticide and their metabolites, which should be the basis for the comprehensive evaluation of pesticides.

**Figure 1:**
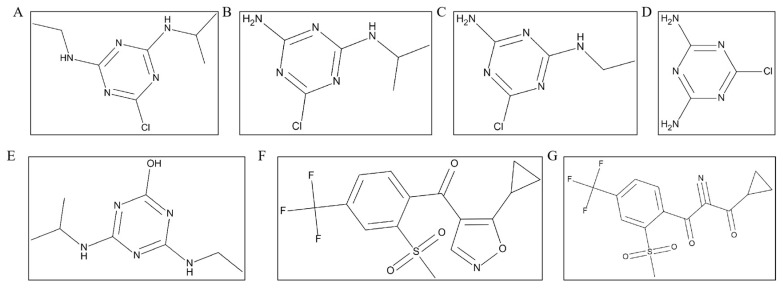
The chemical structural formula of ATR (**A**), DEA (**B**), DIA (**C**), DACT (**D**), HA (**E**), IFT (**F**) and IFT-DKN (**G**).

As far as we know, isoxaflutole (IFT) and atrazine (ATR) have been registered as a mixture in China for controlling annual weeds in corn fields. IFT belongs to the isoxazole pre-emergence herbicides [9]. In plants or the environment, the oxazolium ring of IFT rapidly breaks down to produce diketo-nitrile metabolites (IFT-DKN) that inhibit 4-hydroxyphenylpyruvate dioxygenase (HPPD), which is essential for the biosynthesis of tocopherol and plastoquinone [10,11]. ATR belongs to the triazine herbicides and was first developed by the Swiss company Geigy in 1958 [12]. As a typical photosynthesis inhibitor, ATR occupies a vital position in the herbicide market and is widely used in crops to control annual grasses and broadleaf weeds. ATR forms four primary metabolites: deethylatrazine (DEA), deisopropyl atrazine (DIA), deethyl deisopropyl atrazine (DEDIA), and hydroxyl atrazine (HA) in environments and plants. Their chemical structures are shown in Figure 1. The residue and risk assessment of isoxazolone in plants was defined as the sum of IFT and IFT-DKN. In contrast, ATR was defined as the sum of ATR and its four main metabolites. Herbicides remain in the soil and plants after application in cornfields, potentially impacting consumers’ health through the food chain. In particular, atrazine has been considered a world-recognized endocrine disruptor due to its adverse effects on the biological endocrine system, central nervous system, immune system, and human reproductive development [13,14,15], seriously threatening the ecological environment and consumer health [16]. Therefore, it is necessary to comprehensively study the residues of pesticides and evaluate the dietary risk to guide the safe use of IFT and ATR.

As far as we know, there have been some reports on the residue analysis of ATR, but there are few studies on IFT and its metabolites. The primary detection methods for ATR in environmental and food samples are chromatography, as well as Raman spectroscopy [17] and enzyme-linked immunosorbent assay [18]. Yuan et al. established a gas chromatography–mass spectrometry (GC–MS) method to detect apples, grapes, and tea and a gas chromatography–nitrogen and phosphorus detector (GC–NPD) to quantify ATR residues in soil [19]. Fu et al. established a solid-phase extraction and ultra-high performance liquid chromatography–tandem mass spectrometry (SPE–UPLC–MS/MS) method for detecting ATR in water [20]. Tandon et al. showed a technique for determining ATR in corn kernels, straw, and soil by liquid chromatography with a UV detector [21]. It has been reported that IFT and IFT-DKN in environmental and food samples were determined by chromatography. Lin et al. [6] developed high-performance liquid chromatography–UV (HPLC–UV) and high-performance liquid chromatography–tandem mass spectrometry (HPLC–MS/MS) method for the analysis of IFT and its two metabolites, IFT-DKN and benzoic acid metabolite (BA) in soil, water and plant samples [22,23]. Lan et al. used modified QuEChERS pre-treatment and high-performance liquid chromatography–tandem mass spectrometry to determine isoxazole and its metabolites in corn and straw, and the half-lives of IFT in Shandong and Anhui provinces were 36.4 and 42.1 days, respectively [24]. IFT can effectively kill atrazine-resistant weeds, so the mixture of IFT and ATR has broad application prospects. However, there is no report on the simultaneous detection of IFT, ATR, and their metabolites in fresh corn, corn kernels, and corn straw. Moreover, the terminal residues and dietary risks of IFT and ATR remain to be studied.

In our study, a modified QuEChERS pre-treatment and ultra-high performance liquid chromatography–tandem mass spectrometry (HPLC-MS/MS) methods were developed for the detection of seven target compounds (including two active ingredients and five metabolites) to study terminal residues of, and safety risk assessment for, IFT and ATR. A supervised field experiment was conducted on cornfield under good agricultural practice (GAP) conditions to study the end residues of IFT and ATR in fresh corn, corn kernel, and corn straw. We also assessed the dietary risk of IFT and ATR residues based on pesticide residue data, food consumption, and toxicology data. This work will provide reasonable recommendations for the safe use of IFT and ATR.

## 2. Results

### 2.1. Optimization of UPLC–MS/MS Analysis

Firstly, the standard solutions of 0.1 mg L^−1^ IFT, ATR and their metabolites were scanned in ESI positive ion and negative ion modes, respectively. Considering that a strong response can only be obtained in the negative mode, the negative ion mode was selected for the subsequent analysis of IFT-DKN. On the contrary, the other six compounds can only obtain strong effective peaks in the positive ion mode. Next, the standard solution was scanned in the *m/z* range of 50–400, and the strongest peak found by IFT-DKN was observed at *m/z* = 358.07, corresponding to (M – H). The strongest peaks for the other six compounds corresponded to (M + H). Next, the cone voltage, collision energy and fragmentation were determined using the instrument’s optimization software. The optimized mass spectrometry parameters are shown in Table 1.

**Figure 2:**
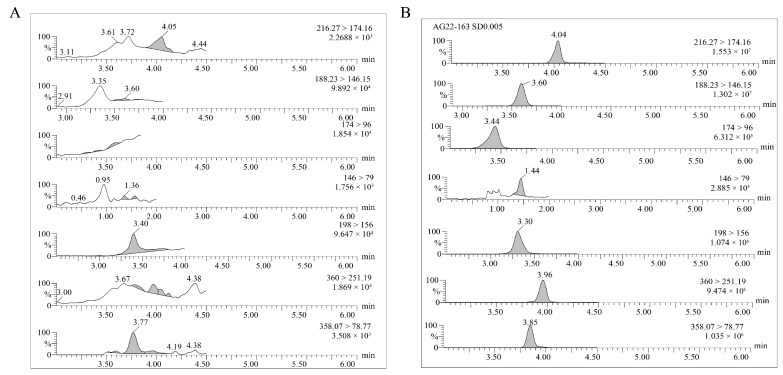
Typical chromatograms of ATR, IFT and their metabolites. (**A**) Blank fresh corn; (**B**) blank fresh corn spiked with 0.005 mg kg^−1^ mixed standard solution.

To obtain optimal chromatographic peak shapes during HPLC–MS/MS analysis, different mobile phase compositions were tested. Three mobile phases, including (A) water/methanol (B) 0.1% water/methanol and (C) 0.2% water/methanol solution, were tested. The results show that adding 0.2% formic acid aqueous solution can effectively improve the sensitivity. Therefore, 0.2% formic acid water/methanol was used as the optimal mobile phase, and good peak shapes were obtained for seven compounds (Figure 2).

### 2.2. QuEChERS Pre-Treatment

The QuEChERS method was developed by Professor Anastassiades in 2003 and includes three steps: extraction, salting out, and purification [25]. It has been widely used to detect single and multiple pesticide residues [26,27]. Acetone, methanol, and ethyl acetate are common solvents used to extract pesticides from different matrices. However, acetone and methanol are miscible with water and difficult to separate, making it impossible to completely transfer pesticides to the organic phase [28]. Ethyl acetate is partially compatible with water, which is not conducive to extracting polar pesticides. As a QuEChERS solvent, acetonitrile has good extraction efficiency for most pesticides. The extract contains fewer interfering substances and is easily separated from water [29]. Therefore, acetonitrile was selected as the extraction solvent in this study. C18 and GCB are commonly used adsorbents in the QuEChERS method. C18 easily adsorbs non-polar substances, such as fats, sterols, and volatile oils; GCB is a regular polyhedron with a uniform graphitized surface and has excellent adsorption performance for samples with high pigment content. Cui et al. used C18 and GCB as purification materials for the QuEChERS method to analyze tembotrione and its metabolite in corn, corn oil, and animal-source foods [30]. Similarly, Zhong et al. used the Quechers method using C18 and GCB as purifiers to extract flumetsulam and florasulam from fresh corn, corn kernels, straw, and soil [31]. Therefore, C18 and GCB were selected as purifying agents.

### 2.3. Method Validation

According to OECD guidelines [32], the accuracy, precision, linearity, matrix effect (ME) and limit of quantitation (LOQ) of the method were verified.

The linear equation and ME of ATR, IFT and their metabolites are shown in Table 2. In the concentration range of 0.0025–0.25 mg kg^−1^, all compounds had good linearity, with the coefficient of determination (R^2^) greater than 0.9912. The chromatograms of blank fresh corn and fresh corn spiked with standard mixed solution (0.005 mg kg^−1^) are shown in Figure 2.

Matrix effect (ME) is an inherent aspect of the ESI source caused by impurities in the sample, which interferes with the quantitative accuracy of target compounds [33]. The ME of ATR and its four metabolites in fresh corn range from −9.1% to 12.7%; the ME of ATR, DEA, DIA and IFT in corn straw were −24.3%, −48.5%, −33.7% and −46.5%, respectively; and the ME of seven compounds in corn kernels vary greatly, with the lowest IFT-DKN being only 0.5% and the highest DACT being 62.9%. Since the ME of all compounds cannot be ignored, matrix-matched standard curves were used for quantification.

**Figure 3:**
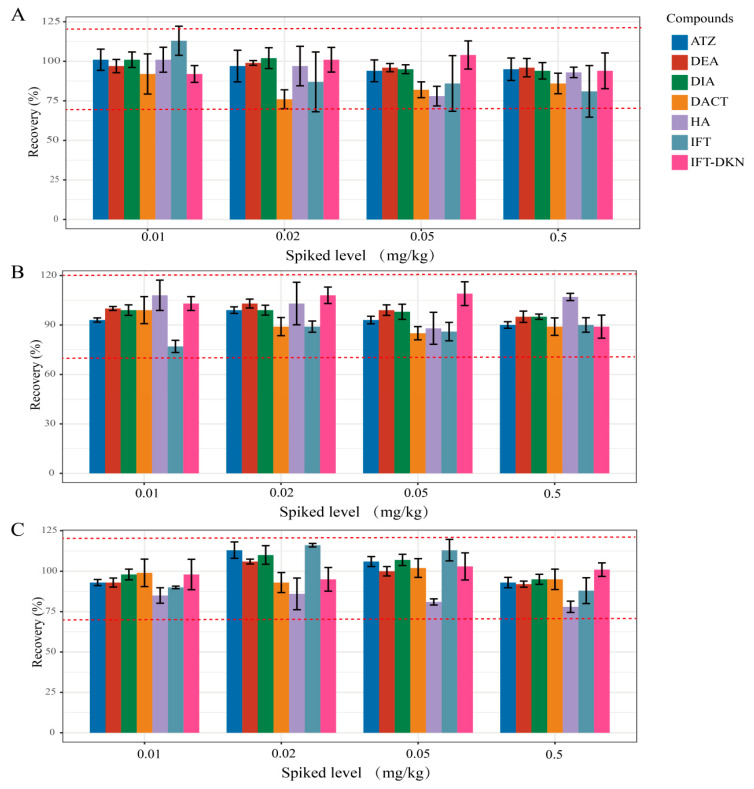
The recoveries and relative standard deviations of ATR, IFT and their metabolites in fresh corn (**A**), corn kernels (**B**) and corn straw (**C**) samples (n = 5).

To evaluate the accuracy and precision, the mixed standards of ATR, IFT and their metabolites were spiked to the blank extracts of fresh corn, corn kernels and corn straw at 0.01, 0.02, 0.05 and 0.5 mg kg^−1^ levels, respectively. The results are shown in Figure 3. The average recoveries of ATR in fresh corn, corn kernels and corn straw were 94–101%, 90–99% and 93–113%, respectively, and the average recoveries of DEA in the three substrates were 96–99%, 95–103% and 92–106%, respectively. The average recoveries of DIA were 94–102%, 95–99% and 95–110%, respectively. The recoveries of DACT were 76–92%, 85–99% and 93–102%, respectively. HA were between 78 and 101%, 88 and 108% and 78 and 86%. The average recovery of IFT in fresh corn, corn kernels and corn straw is 81–113%, 77–90% and 88–116%, respectively. The average recovery of IFT-DKN in fresh corn was 92–104%, in corn kernels 89–109%, and in corn straw 95–103%. For fresh corn, corn kernels and corn straw, the LOQ of these compounds was all 0.01 mg kg^−1^. The recoveries of seven compounds in fresh corn, corn grain and straw were in the range of 76 to 116%, with the relative standard deviations of all compounds ranging from 0.8% to 18.9%, which meets the requirements of OECD guidelines (SANTE 11312/2021) and the guideline for the testing of pesticide residues in crops (NY/T 788–2018).

In conclusion, the method established in this study was reliable, sensitive, has a short sample pre-treatment time and low cost, and can be used to quantify ATR, IFT and their metabolites in fresh corn, corn kernels, and corn straw.

### 2.4. Terminal Residue

The terminal residues of pesticides in food affect food safety and therefore arouse great concern among consumers [34]. The mixture of IFT and ATR has a broad application prospect because of their well-known complementary activity [35,36]. However, the terminal residues of these two compounds and their metabolites in corn fields have not been reported. According to the recommended dosage (active ingredient 874.5 g hm^−2^), 53% isoxaflutole·atrazine suspending agent was sprayed during the corn three-leaf stage, and the final residues of total ATR and IFT in 6 provinces were detected, as shown in Table 3.

The residues of ATR, IFT and their metabolites in fresh corn and corn kernels collected at harvest were all below the LOQ (0.01 mg kg^−1^), and he terminal residue of ATR in corn straw was in the range of <0.05 to 0.17 mg kg^−1^. This herbicide was sprayed at the seedling stage of corn [37], which may be the main reason for the low residues of atrazine, isoxazotrione and their metabolites. Tandon et al. [21] reported that, when atrazine was sprayed before emergence, the atrazine in corn kernels, soil and straw during harvest was less than 0.005 mg kg^−1^. According to the research results of Su et al. [33], the herbicide tembotrione residues in 10 field corn kernel samples were all less than 0.02 mg kg^−1^. Field experiments in four provinces of China showed that the residues of glyphosate and glyphosate in corn grains were lower than 0.09 mg kg^−1^ [38]. Similarly, Zhong et al. [31] reported that the concentration levels of flumetsulam and florasulam in fresh corn and corn kernels during harvest were lower than 0.005 mg kg^−1^. These results demonstrate that herbicides sprayed during the seedling stage or pre-emergence generally have low residue levels in corn kernels. In China, MRL values of IFT in corn and fresh corn have been established to be 0.02* mg kg^−1^, and ATR in corn to be 0.05 mg kg^−1^ [39]. The MRL values of ATR in fresh corn developed by the United States, Japan, and Australia were 0.1, 0.2, and 0.1* mg kg^−1^, respectively. It is safe to spray 53% isoxaflutole/atrazine suspending agent according to the recommended dosage in corn field during harvest.

### 2.5. Chronic Dietary Risk Assessment

The presence of pesticide residues in food may pose risk to humans. Dietary risk assessment is a necessary means to quantify the risk of pesticides in food and guide the safe use of pesticides [40,41]. The dietary risk assessment was calculated based on consumers’ toxicological data, residue levels, and dietary intake. The ADI of ATR and IFT were all 0.02 mg kg^−1^ bw. Considering the risk maximization principle [34], ATR and IFT risk assessment used 0.05 mg kg^−1^ and 0.02 mg kg^−1^ as residual values to calculate NEDI and RQc, respectively. The results of intake risk are shown in Table 4. ATR and IFT are mainly used for weeding in corn fields in China. The chronic dietary risk was assessed based on the corn consumption of men and women of different age groups in the *2010–2013 Monitoring Report on Nutrition and Health Status of Chinese Residents* and the body weight provided in the 2014 National Physical Monitoring Bulletin. For all populations, the range of NEDI of ATR was 3.71 × 10^−6^–1.48 × 10^−5^ mg kg^−1^ bw day^−1^, and that of IFT was 1.48 × 10^−6^–5.91 × 10^−6^ mg kg^−1^ bw day^−1^. The RQc of ATR and IFT were in the range of 0.0074% to 0.0739%, much lower than 100%. These results suggest that the chronic risk associated with ingesting ATR and IFT through maize was acceptable. In addition, the chronic risk of ATR was higher than that of IFT. Regarding chronic dietary risks in different populations, consistent with previous studies [37], the results indicated that the intake risk for children (2–3 years old) was the highest, with increase of age the risk decreased gradually, and the intake risk was the lowest between 30 and 44 years old; for different genders, the risk for women was generally higher than that for men. These results were consistent with previous reports highlighting differences in the risk of dietary pesticide intake by age and sex [42,43], suggesting that certain populations were more susceptible to atrazine, isoxazotrione, and their metabolism through the corn, and subject to health risks.

## 3. Materials and Methods

### 3.1. Chemicals and Reagents

Certified standard IFT (purity 99.84%), ATR (99.37%), DEA (purity 99.06%), DIA (purity 99.02%), DACT (purity 99.14%) and HA (purity 97.24%) were provided by DrEhrenstorfer Ltd. (Augsburg, Germany). IFT-DKN (99.7% purity) was purchased from Beijing Qincheng Yixin Technology Development Co., Ltd. (Beijing, China). HPLC-grade acetonitrile, methanol, and formic acid were purchased from Thermo Fisher Technology Co., Ltd. (Shanghai, China). Tedia Company, Inc., Fairfield, OH, USA, provided analytical acetonitrile. Analytical anhydrous magnesium sulfate and sodium chloride were purchased from Sinopharm Chemical Reagent Co., Ltd. (Shanghai, China). Graphitized carbon black (GCB, 38–120 μm) and octadecyl silane (C18, 50 µm) were purchased from Tianjin Bona Eijer Technology Co., Ltd. (Tianjin, China). PTFE film needle filter (0.22 µm) was purchased from Tianjin Bonaigel Technology Co., Ltd. (Tianjin, China). Purified water was provided by Guangzhou Watsons Food & Beverage Co., Ltd. (Guangzhou, China).

### 3.2. Preparation of Solvent Standard and Matrix Matching Standard Solution

The standard solutions of ATR, DEA, DIA, DACT, and HA of 1000 mg L^−1^ were dissolved to 10 mL with methanol in a brown volumetric flask. The acetonitrile diluted 1000 mg L^−1^ standard solution of 0.0010 g IFT, and IFT-DKN was obtained similarly. The above standard solutions of 1 mL were measured in a 10 mL volumetric flask to prepare the mixed standard solutions of ATR, IFT, and their metabolites of 100 mg L^−1^. The mixed standard solutions were diluted with acetonitrile, fresh corn, corn kernels, and corn straw to prepare different concentrations (0.0025, 0.005, 0.0125, 0.025, 0.125, 0.25 mg L^−1^) and matrix-matching standard solutions. The prepared solution is stored in a refrigerator at 4 °C.

### 3.3. Field Experiment Design and Sampling

According to the guidelines for the Detection of Pesticide residues in crops (NY/T 788, 2018) issued by the Ministry of Agriculture and villages of the People’s Republic of China, field experiments were conducted from May to October 2022 in Shenyang City, Liaoning Province, Harbin City, Heilongjiang Province, Hohhot City, Inner Mongolia, Jinzhong City, Shanxi Province, Changping District, Beijing and Maile City, Honghe Prefecture, Yunnan Province to study the residues of ATR, IFT and their metabolites in corn field. The soil pH value of these plots is between 6.5 and 8.66; the organic matter content is less than 2.9%; the average temperature during the experiment period is between 18.9 and 25.7 °C, and the rainfall is less than 367.4 mm. The commercial pesticide product 53% isoxaflutole·atrazine suspension consists of the active ingredients atrazine (48%) and isoxaflutole (5%). According to Good Agricultural Practice (GAP), 53% isoxaflutole·atrazine suspending agent were sprayed once at the three-leaf stage of corn with an active ingredient of 874.5 g hm^−2^. Each treatment was carried out in a 100 m^2^ plot, repeated twice, and separated by a buffer zone of 0.5 m. At the same time, the same volume of water was sprayed on the control plot.

During harvest, 12 (more than 2 kg) fresh corn, corn kernels, and corn straw samples with normal growth and disease-free were picked randomly from each plot. The fresh corn samples without bracts and filaments were divided into three equal-length segments. The upper, middle, and lower segments were taken respectively, and then the corn segments were crushed and thoroughly mixed. The corn kernel samples without bracts and filaments were threshed, and the grains were well mixed. The corn straw samples in the field were divided into three equal-length segments, and four upper, middle, and lower segments were chopped and thoroughly mixed. Fresh corn, corn kernels, and corn straw were divided into two samples of not less than 200 g; one was the experimental sample, and the other was the backup sample. The collected samples were packed in a sealed pocket, labeled, and stored in cold storage at less than −18 °C for further analysis.

### 3.4. Residue Determination

#### 3.4.1. Extraction and Purification Procedures

Homogenized fresh corn (5.00 g), corn kernels (5.00 g) and corn straw (2.50 g) were weighed into 50 mL centrifuge tubes, respectively. Add 2.5 mL purified water and 10 mL acetonitrile to the centrifuge tube. Cover and shake vigorously for 10 min. Then, add 2 g anhydrous magnesium sulfate and 2 g sodium chloride into the test tube and shock again for 5 min. Centrifuge at 4000 rpm for 5 min. Then, 1.5 mL of the supernatant was transferred to a 2 mL purification tube containing different adsorbent materials. The purification tubes of fresh corn and corn kernels were filled with 150 mg anhydrous magnesium sulfate and 75 mg C18. In comparison, the corn straw purification tubes were filled with 150 mg anhydrous magnesium sulfate, 50 mg C18, and 3.75 mg GCB. The purified tube was swirled for 1 min and centrifuged at 4000 rpm for 5 min. The extracts were filtered through a 0.22 μm syringe and then transferred to an automatic sampler for HPLC-MS/MS analysis.

#### 3.4.2. Instrumental Parameters

The compounds were analyzed by high performance liquid chromatography tandem mass spectrometry (HPLCI-ClassXEVOTQ-XS, Waters, Milford, CT, USA). The chromatographic separation was performed on an ACQUITYUPLC ^®^BEHC18 column (2.1 × 100 mm, 1.7 μm). The mobile phase was (A) 0.2% formic acid aqueous solution and (B) methanol. The flow rate was 0.3 mL min^−1^. The temperature of column box was 40 °C. The injection volume was 5 μL. The conditions of gradient elution were as follows: 10% B (0–2 min), increased to 70% B (2–2.5 min), kept at 70% B (2.5–5.5 min), decreased to 10% B (5.5–5.6 min), and maintained (5.6–6 min). Except IFT-DKN (negative), other compounds were monitored by mass spectrometry with positive ionization mode. Data analysis was performed using MassLynx 4.0 software.

#### 3.4.3. Method Validation

The method’s accuracy, precision, linearity, ME and LOQ were validated. Recovery experiments were performed by spiking ATR, IFT and their metabolites to blank samples at concentrations of 0.01, 0.02, 0.05, and 0.5 mg kg^−1^, respectively, and each was repeated five times. Solvent or matrix-matched standard solutions with concentrations ranging from 0.0025 to 0.25 mg kg^−1^ were serially diluted. The limit of quantitation (LOQ) was determined as the lowest spiked level.

Matrix effects (ME) were assessed by comparing the slope of the matrix-matched standard to the acetonitrile standard. The calculation method was:

(1) $$ME\left(\%\right)=\frac{\left(Smatrix-Ssolvent\right)}{Ssolvent}\times 100\%$$

where, S_matrix_ and S_solvent_ were the slopes of the matrix matching standard and solvent standard, respectively. ME can be ignored if the ME value was within the range of −20–20%. Otherwise, a matrix enhancing or attenuating effect was exhibited.

### 3.5. Terminal Residue and Dietary Risk Assessment

#### 3.5.1. Residue Definition

According to the residue definition of risk assessment (JMPR, 2013), the total residue of IFT was calculated according to the following equation:

C_IFT_ = C_IFT_ + C_IFT-DKN_ (2)

According to the Pesticide Registration Residue Test Residues and the Catalog of Residues for Dietary Risk Assessment in Foods of Plant Origin, the total residues of ATR were estimated with the following equation:

C_ATR_ = C_ATR_ + C_DEA_ × 1.15 + C_DIA_ × 1.24 + C_DACT_ × 1.48 + C_HA_ × 1.09 (3)

where C_IFT_, C_IFT-DKN_ C_ATR_, C_DEA_, C_DIA_, C_DACT_ and C_HA_ are the concentrations of IFT, IFT-DKN, ATR, DEA, DIA, DACT and HA. 1.15, 1.24, 1.48 and 1.09 are the molecular weight ratios of DEA, DIA, DACT and HA to ATR, respectively. If the residues of IFT, ATR and their metabolites were lower than their limited limits (LOQ), the value of LOQ was considered directly, and the C_sum_ was calculated directly based on the sum of LOQ values of all compounds.

#### 3.5.2. Dietary Risk Assessment

The risk quotient (RQ_c_) was used to assess further the risk of chronic dietary intake of IFT and ATR and was calculated according to the following equation.

NEDI = ∑(STMR × F_i_)/bw (4)

RQ_c_ = NEDI/ADI × 100% (5)

where NEDI (mg kg^−1^ bw day^−1^) is the national estimated daily intake, and STMR (mg kg^−1^) is the standard median residual value. F_i_ (kg) represents the consumption of a given food by a specific population. bw is the average body weight, kg. The ADI of IFT and ATR are both 0.02 mg kg^−1^ bw day^−1^.

## 4. Conclusions

We established an improved QuEChERS pre-treatment and ultra-high performance liquid chromatography–tandem mass spectrometry method to detect ATR, IFT and metabolites. The technique has good linearity, accuracy, and precision. Under GAP conditions, samples of fresh maize, corn kernels, and corn straw after pesticide application were collected in six provinces in China. The results showed that the residues of ATR, IFT, and their metabolites in fresh corn and corn kernels were all lower than LOQ. IFT was also not detected in corn straw, but ATR residue, less than 0.135 mg kg^−1^, was detected. All consumers’ risk quotients (RQ_c_) were below 100%, indicating that the chronic risk of ATR and IFT is acceptable. It should be noted that children (2–3 years old) and women have a relatively higher risk of chronic diseases than other groups.

## Figures and Tables

**Table 1:** Instrument parameters of ATR, IFT and their metabolites in multiple reaction monitoring (MRM) mode.

Compounds	Retention Time (min)	Precursor Ion (m z^−1^)	Product Ion (m z^−1^)	Cone Voltage (CV, V)	Collision Energy (CE, eV)
ATR	4.04	216.27	174.16 *	20	28
			96.14		21
DEA	3.60	188.23	78.9	30	30
			146.15 *		19
DIA	3.44	174.0	96.0 *	65	20
			131.9		15
DACT	1.44	146.0	79.0 *	70	19
			103.99		17
HA	3.30	198.0	156.0 *	30	17
			113.9		21
IFT	3.96	360.0	251.19 *	30	18
			219.9		40
IFT-DKN	3.85	358.07	78.77 *	−20	−17
			278.0		−14

* represents quantitative ion.

**Table 2:** Linear equations, correlation coefficients and matrix effects of all compounds in 0.0025–0.25 mg kg^−1^.

Compound	Matrix	Equation	Determination Coefficient (R^2^)	ME (%)
ATR	Acetonitrile	y = 1.92473 × 10^8^x + 171,768	0.9961	-
	Fresh corn	y = 1.78484 × 10^8^x + 151,436	0.9972	−7.3
	Corn kernels	y = 1.72312 × 10^8^x − 36,266.5	0.9990	−10.5
	Corn straw	y = 1.45725 × 10^8^x + 64,352.9	0.9991	−24.3
DEA	Acetonitrile	y = 1.69395 × 10^8^x + 222,823	0.9912	-
	Fresh corn	y = 1.53907 × 10^8^x + 183,749	0.9945	−9.1
	Corn kernels	y = 1.48905 × 10^8^x − 28,122.8	0.9963	−12.1
	Corn straw	y = 8.71969 × 10^7^x + 8952.86	0.9996	−48.5
DIA	Acetonitrile	y = 1.22314 × 10^7^x + 12,671.5	0.9965	-
	Fresh corn	y = 1.17502 × 10^7^x + 7327.07	0.9981	−3.9
	Corn kernels	y = 1.54207 × 10^7^x + 565.797	0.9986	26.1
	Corn straw	y = 8.11043 × 10^6^x − 1082.61	0.9999	−33.7
DACT	Acetonitrile	y = 92,470.9x + 15.5547	0.9994	-
	Fresh corn	y = 98,205.3x + 21.2445	0.9977	6.2
	Corn kernels	y = 150,640x + 103.791	0.9915	62.9
	Corn straw	y = 99,527x − 4.04451	0.9991	7.6
HA	Acetonitrile	y = 2.51197 × 10^7^x − 13,594.8	0.9995	-
	Fresh corn	y = 2.8312 × 10^7^x + 40,610.5	0.9933	12.7
	Corn kernels	y = 2.74648 × 10^7^x + 43,150.3	0.9912	9.3
	Corn straw	y = 3.17866 × 10^7^x + 49,750.2	0.9906	26.5
IFT	Acetonitrile	y = 1.31221 × 10^8^x + 97,738.5	0.9940	-
	Fresh corn	y = 1.16984 × 10^8^x + 144,660	0.9944	−10.8
	Corn kernels	y = 1.02943 × 10^8^x − 71,139.6	0.9917	−21.5
	Corn straw	y = 7.02378 × 10^7^x − 3031.07	0.9960	−46.5
IFT-DKN	Acetonitrile	y = 1.96818 × 10^7^x − 18,509.5	0.9962	-
	Fresh corn	y = 2.69316 × 10^7^x − 5800.49	0.9994	36.8
	Corn kernels	y = 1.97881 × 10^7^x − 13,759.9	0.9967	0.5
	Corn straw	y = 3.48496 × 10^7^x + 26,353	0.9971	77.1

**Table 3:** Terminal residues of ATR and IFT in fresh corn, corn kernels and corn straw samples.

Location	Total ATR Residue (mg kg^−1^) *			Total IFT residue (mg kg^−1^) *		
	Fresh Corn	Corn Kernels	Corn Straw	Fresh Corn	Corn Kernels	Corn Straw
Liaoning	<0.05	<0.05	0.067 ± 0.0014	<0.02	<0.02	<0.02
Heilongjiang	<0.05	<0.05	<0.05	<0.02	<0.02	<0.02
Neimenggu	<0.05	<0.05	0.11 ± 0.085	<0.02	<0.02	<0.02
Shanxi	<0.05	<0.05	0.071 ± 0.0014	<0.02	<0.02	<0.02
Beijing	<0.05	<0.05	0.135 ± 0.0071	<0.02	<0.02	<0.02
Yunnan	<0.05	<0.05	<0.05	<0.02	<0.02	<0.02

* Total ATR and total IFT represent the sum of ATR and total IFT and their metabolites, respectively.

**Table 4:** Chronic dietary risk assessment for ATR and IFT in a representative population.

Gender	Age (Years)	Average bw (kg)	Fi (kg)	ATR			IFT		
				STMR (mg kg^−1^)	NEDI (mg kg^−1^ bw Day^−1^)	RQ_c_ (%)	STMR (mg kg^−1^)	NEDI (mg kg^−1^ bw Day^−1^)	RQ_c_ (%)
Male	2–3	16.6	0.0047	0.05	1.42 × 10^−5^	0.0708	0.02	5.66 × 10^−6^	0.0283
	4–6	20.6	0.004		9.71 × 10^−6^	0.0485		3.88 × 10^−6^	0.0194
	7–10	31.8	0.0046		7.23 × 10^−6^	0.0362		2.89 × 10^−6^	0.0145
	11–13	46.8	0.0051		5.45 × 10^−6^	0.0272		2.18 × 10^−6^	0.0109
	14–17	59.1	0.0063		5.33 × 10^−6^	0.0266		2.13 × 10^−6^	0.0107
	18–19	63.4	0.0051		4.02 × 10^−6^	0.0201		1.61 × 10^−6^	0.0080
	20–29	68.8	0.0051		3.71 × 10^−6^	0.0185		1.48 × 10^−6^	0.0074
	30–44	71.4	0.0057		3.99 × 10^−6^	0.0200		1.60 × 10^−6^	0.0080
	45–59	70.3	0.0071		5.05 × 10^−6^	0.0252		2.02 × 10^−6^	0.0101
	60–69	67.1	0.0092		6.86 × 10^−6^	0.0343		2.74 × 10^−6^	0.0137
Female	2–3	15.9	0.0047		1.48 × 10^−5^	0.0739		5.91 × 10^−6^	0.0296
	4–6	19.6	0.0042		1.07 × 10^−5^	0.0536		4.29 × 10^−6^	0.0214
	7–10	29.8	0.0048		8.05 × 10^−6^	0.0403		3.22 × 10^−6^	0.0161
	11–13	44.4	0.0058		6.53 × 10^−6^	0.0327		2.61 × 10^−6^	0.0131
	14–17	51.6	0.0046		4.46 × 10^−6^	0.0223		1.78 × 10^−6^	0.0089
	18–19	52.7	0.0069		6.55 × 10^−6^	0.0327		2.62 × 10^−6^	0.0131
	20–29	54.6	0.0069		6.32 × 10^−6^	0.0316		2.53 × 10^−6^	0.0126
	30–44	57.9	0.0064		5.53 × 10^−6^	0.0276		2.21 × 10^−6^	0.0111
	45–59	59.9	0.008		6.68 × 10^−6^	0.0334		2.67 × 10^−6^	0.0134
	60–69	59.5	0.0099		8.32 × 10^−6^	0.0416		3.33 × 10^−6^	0.0166

## Data Availability

The data presented in this study are available from the authors upon request.

## Sample Availability

Samples of the compounds are available from the authors.
